# Illumination and Reflectance Estimation with its Application in Foreground Detection

**DOI:** 10.3390/s150921407

**Published:** 2015-08-28

**Authors:** Gang Jun Tu, Henrik Karstoft, Lene Juul Pedersen, Erik Jørgensen

**Affiliations:** 1 Department of Animal Science, Aarhus University, 8830 Tjele, Denmark; E-Mails: lene.juulpedersen@anis.au.dk (L.J.P.); erik.jorgensen@anis.au.dk (E.J.); 2 Department of Engineering, Aarhus University, 8000 Aarhus C, Denmark; E-Mail: hka@eng.au.dk

**Keywords:** grayscale video recordings, homomorphic wavelet filter, wavelet quotient image model, illumination and reflectance estimation, foreground detection

## Abstract

In this paper, we introduce a novel approach to estimate the illumination and reflectance of an image. The approach is based on illumination-reflectance model and wavelet theory. We use a homomorphic wavelet filter (HWF) and define a wavelet quotient image (WQI) model based on dyadic wavelet transform. The illumination and reflectance components are estimated by using HWF and WQI, respectively. Based on the illumination and reflectance estimation we develop an algorithm to segment sows in grayscale video recordings which are captured in complex farrowing pens. Experimental results demonstrate that the algorithm can be applied to detect the domestic animals in complex environments such as light changes, motionless foreground objects and dynamic background.

## 1. Introduction

Foreground detection is an important preliminary step of many video analysis systems. Many algorithms have been proposed in the last years, but there is not yet a consensus on which approach is the most effective, not even limiting the problem to a single category of videos. A common approach for foreground detection is background subtraction. There are many background removal methods available and the most recent surveys on the methodologies can be found in [1,2,3,4,5,6,7]. It is well known that background subtraction techniques are sensitive two problems [6]: the first is the foreground capturing upon light changes in the environment and the second is motionless foreground objects. Li *et al.* [8] have proposed a method that utilizes multiple types of features (*i.e*., spectral, spatial and temporal features) for modelling complex background. Unfortunately, this method wrongly integrated a foreground object into the background if the object remained motionless for a long time duration [8]. Also, there are some foreground detection methods based on wavelet such as [9,10]. In [9], the authors proposed a discrete wavelet transform based method for multiple objects tracking and identification. Khare *et al.* [10] introduced a method for segmentation of moving object which is based on double change detection technique applied on Daubechies complex wavelet coefficients of three consecutiv frames.

In order to reduces the above problems (*i.e*., light changes and motionless foreground objects) in foreground detection, in this paper, we introduce a method to estimate illumination and reflectance components of grayscale images in video recordings. The method is related to the illumination-reflectance model (IRM) [11] for illumination and reflectance estimation from an observed image. In general, it is difficult to calculate the two components from a real image, since it involves many unknown factors such as description of the lighting in the scene. For any image, Chen *et al.* [12] showed that there are no discriminative functions which are invariant to the illumination. In image processing, the realistic simplified IRM [11] in literature explains an image *f* at a pixel as:

(1) f(x,y)=i(x,y)·r(x,y)

where i(x,y) and r(x,y) stand for the illumination and reflectance components, respectively. (x,y) is the pixel position. Normally, to eliminate the unwanted influences of varying lights, applying a Fourier transform to the logarithmic image, multiplying by a highpass filter, this processing is called homomorphic filtering [13]. When an image is transformed into the Fourier domain, the low frequency component usually corresponds to smooth regions or blurred structures of the image, whereas high-frequency component represents image details, edges and noises. However, it is obvious that any low frequency data in the reflectance will also be eliminated [13]. More recently, many methods have been proposed to improve homomorphic filters such as [14,15] and used for various practical applications. Toth *et al.* [16] presented a method for motion detection, which is based on combining a motion detection algorithm with a homomorphic filter which effectively suppresses variable scene illumination.

In order to estimate the illumination component of an image, we use a homomorphic wavelet filter (HWF) that is based on dyadic wavelet transform (DWT) [17]. Our HWF is applied to improve the accuracy of the illumination estimation which is estimated by the inverse DWT in logarithmic space. To estimate the reflectance component, we define a wavelet-quotient image (WQI) model in intensity space. The WQI model parallels the former idea of a self-quotient image (SQI) model [18]. In the SQI model, the illumination is eliminated by division over a smoothed version of the image. This model is very simple and can be applied to any single image [18]. In the WQI model, the numerator is calculated based on a feature preserved anisotropic filter applied on the original image and the denominator is the coarse image of DWT.

Based on HWF and WQI, we develop an algorithm to segment sows in grayscale video recordings of farrowing pens. The algorithm has five stages: (1) estimate the illumination component of the reference (*i.e*., background) and current images; (2) estimate the reflectance component of the current image;(3) measure the local texture differences between the reference and current images; (4) synthesize a new image based on the above estimating components and the local texture differences, so that background objects are much darker than foreground objects; (5) detect the foreground object (*i.e*., the sow) based on the synthesized image.

Some methods for detecting pigs have been presented in the literature such as [19,20,21,22,23,24,25,26]. But the results for most of these methods have not been discussed in relation to complicated scenes (e.g., light changes and motionless foreground objects). For example, in [19], the major problem during tracking was the loss of tracking due to large, unpredictable movements of the piglets, because the tracking method required the objects to move [19]. Our experimental results demonstrate that our algorithm can be applied to detect the domestic animals in complex environments such as light changes, motionless foreground objects and dynamic background.

The rest of this paper is organized as follows. Section 2 presents the five stages. The proposed algorithm is described in Section 3. Section 4 describes the data that is used in this study. The experimental results are contained in Section 5, and the paper is concluded in Section 6.

## 2. Methodology

The discrete DWT [17] has proved very useful when analysis of multiscale features is important. It can provide a coarse and two detail representations of an image *f* through different scale independent decomposition. The DWT is implemented using halfband lowpass and highpass filters forming a filterbank together with downsamplers. The filterbank produces two sets of coefficients: (1) the orthogonal detail coefficients $W_{2^{j}}^{1}f$ and $W_{2^{j}}^{2}f$ that are the even outputs of the highpass filter; (2) the coarse/approximation coefficients $S_{2^{j}}f$ which are the even outputs of the lowpass filter, where *j* is the multi-resolution level and j∈Z. For a J-level, the following collection is called the 2D discrete DWT:

(2) $${\left\{S_{2^{j}}f,\;\left\{W_{2^{j}}^{1}f,\;W_{2^{j}}^{2}f\right\}\right\}}_{1\leq j\leq J}$$

$S_{2^{j}}$ is defined by $S_{2^{j}}f\left(x,y\right):=f*\phi_{2^{j}}\left(x,y\right)$, where (x,y) is the pixel position and * is the standard convolution, $\phi_{2^{j}}$ is given by $\phi_{2^{j}}\left(x,y\right):=\frac{1}{2^{j}}\phi \left(\frac{x}{2^{j}}\frac{y}{2^{j}}\right)$, where *ϕ* is a scaling function. $W_{2^{j}}^{1}$ and $W_{2^{j}}^{2}$ are defined by: $W_{2^{j}}^{i}f\left(x,y\right)=f*\psi_{2^{j}}^{i}\left(x,y\right)$ (i=1,2). $\psi_{2^{j}}^{i}\left(x,y\right):=\frac{1}{2^{j}}\psi^{i}\left(\frac{x}{2^{j}},\frac{y}{2^{j}}\right)$, where $\psi^{i}$ is a wavelet function. The modulus $M_{2^{j}}$ of the wavelet transform is given:

(3) $$M_{2^{j}}f\left(x,y\right)=\sqrt{|W_{2^{j}}^{1}{f\left(x,y\right)|}^{2}+{\left|W_{2^{j}}^{2}f\left(x,y\right)\right|}^{2}}$$

The reconstructed image is gotten by using the three decomposition components. At each scale $2^{j}$, $S_{2^{j-1}}f$ is reconstructed from $S_{2^{j}}f$, $W_{2^{j}}^{1}f$ and $W_{2^{j}}^{2}f$. The reconstructed image $\tilde{f}$ is $S_{2^{0}}f$. For the details 2D discrete DWT, we refer to [17].

The rest of this section will present the five stages that will be used in our proposed algorithm: Homomorphic wavelet filter; Wavelet-quotient image model; Texture difference measure; The synthesizing image and Foreground detection.

### 2.1. Homomorphic Wavelet Filter

In logarithmic space, the IRM in Equation (1) can be rewritten F=I+R, where the symbols are defined as below:

(4) F(x,y)=logf(x,y)

(5) I(x,y)=logi(x,y),R(x,y)=logr(x,y)

where log means logarithmic. In general, the illumination of a scene varies slowly over space, whereas the reflectance component contains mainly spatially high-frequency details.

**Figure 1:**
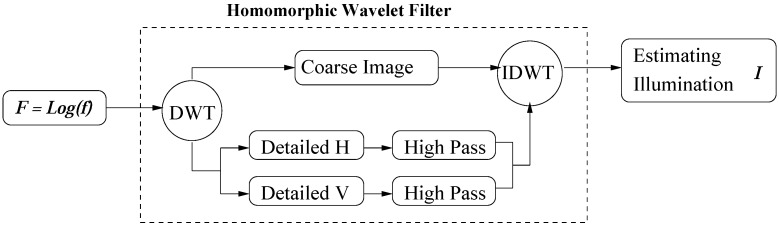
The flow chart of our homomorphic wavelet filter. DWT: dyadic wavelet transform analysis; IDWT: inverse dyadic wavelet transform; Detailed H: $W_{2^{j}}^{1}$ and Detailed V: $W_{2^{j}}^{2}$; High Pass: Butterworth filter H; *Log* means logarithmic.

In this paper, we use a homomorphic wavelet filter (HWF), which is used to estimate the illumination component *I* in logarithmic space. In our HWF, the discrete DWT takes the place of Fourier transform. Our HWF is somewhat similar to [27] and its flowchart is shown in Figure 1. During DWT decomposition process across different scales, the illumination component of an image is well preserved while the shape variation against other individuals is largely reduced. Therefore, the approximation coefficients $S_{2^{J}}F$ in the scale gives a good approximate of the illumination component *I* (*i.e*., the coarse coefficients $S_{2^{J}}F$ contains almost all the illumination component of the image *F*). Then a homomorphic filtering procedure is performed to filter out the small amount of illumination component distributed in all the detail coefficients $W_{2^{J}}^{1}F$ and $W_{2^{J}}^{2}F$. The three decomposed parts are combined together and the inverse DWT (IDWT) is performed to get the final estimate $\tilde{I}$ of the illumination in the image *F*:

(6) $$\tilde{I}=\mathrm{IDWT}\left(S_{2^{J}}F+\left(W_{2^{J}}^{1}F\right)\cdot H+\left(W_{2^{J}}^{2}F\right)\cdot H\right)$$

where H is a high-pass Butterworth filter given by:

(7) $$H\left(u,v\right)=\frac{1}{1+{\left[\frac{D_{0}}{D(u,v)}\right]}^{2n}}$$

where $D_{0}$ is the cutoff amplitude in wavelet domain, *n* is the order of filter and D(u,v) is the amplitude at location (u,v):

(8) $$D\left(u,v\right)=\sqrt{{\left(u-\frac{M}{2}\right)}^{2}+{\left(v-\frac{N}{2}\right)}^{2}}$$

where M×N is the size of image. We use J=3, $D_{0}=0.95$ and n=2 as default setting in our experiments.

### 2.2. Wavelet-Quotient Image Model

In order to extract the reflectance component from an image, we define a wavelet-quotient image (WQI) model that is similar to a self-quotient image (SQI) model [18,28], which is proposed based on the basic conception of the quotient image model [29]. The SQI implements the normalization by dividing the low-frequency part of the original image and generates the reflectance component (*i.e*., illumination invariant) features:

(9) $$\mathrm{SQI}\left(x,y\right)=\frac{f(x,y)}{S(x,y)}=\frac{f(x,y)}{L(x,y)*f(x,y)}$$

where S(x,y) denotes the low-frequency component, which is computed as the convolution between a smoothing filter L(x,y) and the original image f(x,y). Since the features belonging to the low-frequency bands are removed, then *SQI* contains the illumination invariant features. The *SQI* method neither uses the information about the lighting source, nor needs a training set, and directly extracts the illumination invariant features. It is a very simple model and can be applied to any single image.

In our WQI model, the numerator image should be smoothed by a feature preserved anisotropic filter that can extract features effectively, since an anisotropic filter smooths the image in homogeneous area but preserves edges and enhances them. In this paper, the Perona-Malik diffusion model [30,31] is used, because this diffusion model is a method aiming at reducing image noise without removing significant parts of the image content, typically edges or other details that are important for the interpretation of the image.

The denominator of WQI is the coarse coefficients $S_{2^{J}}f\left(x,y\right)$ of the DWT at final level *J* which correspond to the low-frequency of the image *f* (see Equation (2)). The DWT allows the image decomposition in different kinds of coefficients, while preserving the image information without any loss.

**Definition:** *The wavelet-quotient image* WQI *of a gray-level image f is defined by:*

(10) $$\mathrm{WQI}\left(x,y\right)=\frac{\mathrm{Diffuse}\left(f(x,y)\right)}{S_{2^{J}}f\left(x,y\right)}=\frac{\mathrm{Diffuse}\left(f(x,y)\right)}{\phi_{2^{J}}*f\left(x,y\right)}$$

where $\phi_{2^{J}}$ is given above and * is the standard convolution; $\mathrm{Diffuse}\left(f(x,y)\right)$ is the diffused image of *f* by using the Perona-Malik diffusion model. In the DWT schema, the decomposition is recursively performed over the coarse image.

### 2.3. Texture Difference Measure

In order to measure texture differences between two images, we use the idea which is proposed in [32]. This idea is based on the gradient value of each pixel. A good texture difference measure should be able to represent the difference between two local spatial gray level arrangements accurately [32]. Since the wavelet detail coefficients (see Equation (2)) are a good measure to describe how the gray level changes within a neighbourhood [17], so it is less sensitive to light changes and can be used to derive an accurate local texture difference measure. Therefore, the gradient vector $f^{{}^{\prime }}\left(p\right)$ in this paper is defined as below:

(11) $$f^{{}^{\prime }}\left(p\right)=\left(W_{2^{J}}^{1}f\left(p\right),W_{2^{J}}^{2}f\left(p\right)\right)$$

where the position of the pixel *p* is (x,y).

The module magnitude difference (MMD) of the images $f_{1}$ and $f_{2}$ at point *p* can be defined:

(12) $${\mathrm{MMD}}_{f_{1},f_{2}}\left(p\right)=\left|M_{2^{J}}f_{1}\left(p\right)-M_{2^{J}}f_{2}\left(p\right)\right|$$

where $M_{2^{J}}f_{1}\left(p\right)$ and $M_{2^{J}}f_{2}\left(p\right)$ are given by Equation (3). The cross correlation (CC) of gradient vector of images $f_{1}$ and $f_{2}$ at each point *p* can be defined:

(13) $${\mathrm{CC}}_{f_{1},f_{2}}\left(p\right)=f_{1}{}^{\prime }\left(p\right)\cdot f_{2}{}^{\prime }\left(p\right)=M_{2^{J}}f_{1}\left(p\right)\cdot M_{2^{J}}f_{2}\left(p\right)\cdot \cos\theta$$

where *θ* is the angle between the two vectors $f_{1}{}^{\prime }\left(p\right)$ and $f_{2}{}^{\prime }\left(p\right)$ that are defined in Equation (11). The texture difference rate (TDR) of the two images at point *p* can be defined:

(14) $${\mathrm{TDR}}_{f_{1},f_{2}}\left(p\right)=1-\frac{2\cdot \sum_{q\in N_{p}}{\mathrm{CC}}_{f_{1},f_{2}}\left(q\right)}{\sum_{q\in N_{p}}\left(M_{2^{J}}^{2}f_{1}\left(q\right)+M_{2^{J}}^{2}f_{2}\left(q\right)\right)}$$

where $N_{p}$ denotes the 3 × 3 neighbourhood centred at *p*. As discussion in [32], ${\mathrm{TDR}}_{f_{1},f_{2}}\left(p\right)\approx 0$ when the texture of point *p* doesn’t change between the two corresponding images.

### 2.4. The Synthesizing Image

After the illumination components of both the current (*subscript c*) and reference (*subscript r*) images are estimated by using the HWF, we need to synthesize a virtual image $f_{c}^{\mathrm{syn}}$ by using the estimating illumination ${\tilde{I}}_{r}$ of the reference image, and the estimating illumination ${\tilde{I}}_{c}$ and reflectance ${\tilde{r}}_{c}$ of the current image:

(15) $$f_{c}^{\mathrm{syn}}=\exp\left(\mathrm{Mapping}\left({\tilde{I}}_{c},{\tilde{I}}_{r}\right)+\log\left({\tilde{r}}_{c}\right)\right)$$

where exp is exponential function, ${\tilde{I}}_{c}$ and ${\tilde{I}}_{r}$ are given by Equation (6), and ${\tilde{r}}_{c}$ is the corresponding *WQI* which is given by Equation (10). The “Mapping” function is defined by:

(16) $$\mathrm{Mapping}\left({\tilde{I}}_{c},{\tilde{I}}_{r}\right)\left(p\right)=\alpha {\tilde{I}}_{c}\left(p\right)-{\tilde{I}}_{r}\left(p\right)$$

where *α* is given by:

(17) $$\alpha =\left\{\begin{array}{cc} c, & if\;{\mathrm{TDR}}_{f_{c},f_{r}}\left(p\right)>\gamma \\ 1, & otherwise \end{array}\right.$$

where c>1 and ${\mathrm{TDR}}_{f_{c},f_{r}}\left(p\right)$ is given by Equation (14). In our implementation, c=2. Based on [32], we choose the threshold *γ* as 0.5 that corresponds to the texture change (*i.e*., ${\mathrm{TDR}}_{f_{c},f_{r}}\left(p\right)\leq 0.5$, the texture does not change). According to the above definitions, the synthesizing image $f_{c}^{\mathrm{syn}}$ will give the foreground objects more brightness, *i.e*., the background objects will be much darker than the foreground objects in $f_{c}^{\mathrm{syn}}$.

### 2.5. Foreground Detection

In order to detect the foreground objects, we use a simple *k*-means [33] technique on the synthesize image (*i.e*., Equation (15)) to classify the pixels into three clusters (*i.e*., k=3). The foreground objects are extracted by using

(18) $$f_{c}^{\mathrm{fg}}\left(p\right)=\left\{\begin{array}{cc} 1, & if\;p\;\mathrm{belongs}\mathrm{to}\mathrm{the}\mathrm{highest}\mathrm{level}\mathrm{of}\mathrm{the}\mathrm{clusters}(i.e.,\;p\;\mathrm{is}\mathrm{the}\mathrm{foreground}\mathrm{object})\\ 0, & otherwise \end{array}\right.$$

Since the textures of foreground and background objects have a significant difference in our application, so it is easy to extract the boundaries $f_{c}^{\mathrm{bou}}$ of foreground objects by using MMD which is calculated by Equation (12):

(19) $$f_{c}^{\mathrm{bou}}\left(p\right)=\left\{\begin{array}{c} 1,\;\mathrm{if}\;{\mathrm{MMD}}_{f_{c},f_{r}}\left(p\right)>M_{th}\\ 0,\;\mathrm{otherwise} \end{array}\right.$$

where $M_{th}$ is a threshold.

In order to segment the sow, we combine the two binary images $f_{c}^{\mathrm{fg}}$ (Equation (18)) and $f_{c}^{\mathrm{bou}}$ (Equation (19)). We do the following steps using the two binary images:

1. A morphological close filtering is performed on the image $f_{c}^{\mathrm{bou}}$ using a circular structuring element of 3-pixel diameter to fill the gaps and smooth out the edges.
2. To separate the piglets and the sow, we subtract the two images: $f_{c}^{\mathrm{sub}}=∥f_{c}^{\mathrm{fg}}-f_{c}^{\mathrm{bou}}∥$. After subtracting, the piglets and the sow are separated if they connect together. We remove the small areas in the image $f_{c}^{\mathrm{sub}}$, so the piglets are eliminated. Then, the area of the foreground object, which is a total number of pixels of the sow in the image $f_{c}^{\mathrm{sub}}$, is extracted.
3. Now we combine the images $f_{c}^{\mathrm{sub}}$ and $f_{c}^{\mathrm{bou}}$: $f_{c}^{\mathrm{com}}=f_{c}^{\mathrm{sub}}+f_{c}^{\mathrm{bou}}$. After combining, again, in order to eliminate the boundaries of the piglets, we remove the small areas in the image $f_{c}^{\mathrm{com}}$.
4. The connected components algorithm and some other post-processing operations are performed in the combined image $f_{c}^{\mathrm{com}}$ to extract the shape of the sow.

## 3. The Proposed Algorithm

**Figure 2:**
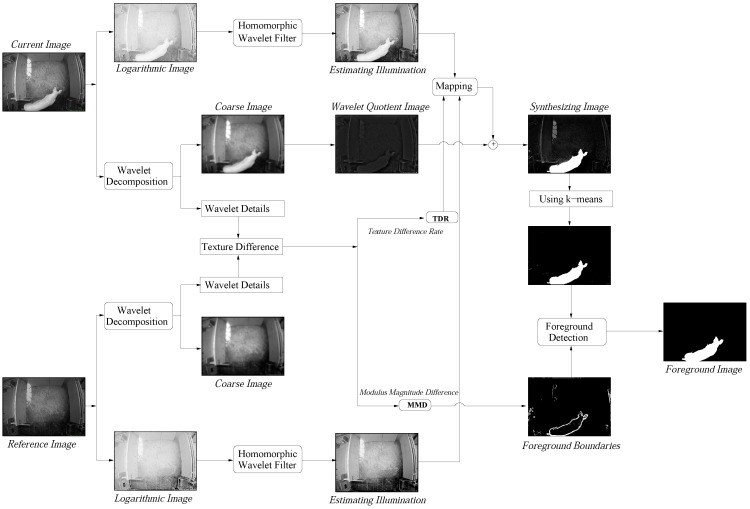
The flowchart of our algorithm. Three images are used to synthesize: two estimating illumination images and a wavelet quotient image (*i.e*., the reflectance component of the current image).

Now we are ready to describe our algorithm using the methods which are presented in the previous section. The flowchart and pseudocode of the algorithm are shown in Figure 2 and Table 1, respectively. In Table 1, the illumination component is estimated in logarithmic space and the reflectance component is estimated in intensity space.

It is observed that a large amount of computation for the proposed algorithm (see Figure 2). The complexity for the 2D-discrete DWT and its inverse are highest in our algorithm. Although the filters of the spline wavelet are short, the complexity for decomposition and reconstruction require $O\left(N^{2}\log\left(N\right)\right)$ [17] for an image of size N×N. Hence, the 2D-discrete DWT is still very computing intensive. Moreover the representation has $\left(2j+1\right)N^{2}$ values, which must be stored in memory. With a 3.0-GHz Pentium CPU PC, real-time processing of image sequences is achievable at a rate of about 1 frames per second for gray images sized 768 × 576. In our implementation, all codes are implemented using Matlab. If it is implemented by C++, the speed will be improved.

**Table 1:** The pseudocode of our algorithm.

0. Input the reference image (*i.e*., background);
Estimate the illumination of the reference image by using HWF (see Figure 1);
Estimate the coarse image and wavelet details of the reference image by using Equation (2);
1. **repeat**
2. Input the current image;
3. Estimate the illumination of the current image by using HWF (see Figure 1);
4. Estimate the coarse image and wavelet details of the current image by using Equation (2);
5. Estimate the reflectance of the current image by using WQI (*i.e*., Equation (10));
6. Synthesize an image by using Equation (15), the estimated illumination and reflectance of the current image and the estimated illumination of the reference image are used in the function;
7. Estimate the foreground objects based on the synthesizing image by using Equation (18);
8. Estimate the boundaries of the foreground objects by using Equation (19);
9. Detect the sow based on the foreground (*i.e*., step 7) and boundary (*i.e*., step 8) images, the approach is described in Section 2.5.
10. **end**

Especially in real-time applications, the general purpose processors could not deliver the necessary performance for the computation of the 2D discrete DWT. A fast implementation is therefore obvious. If the hardware of parallel architecture such as GPU is employed, the computation performance of the DWT could be significantly improved [34]. Hence, our algorithm is able to run so fast in real-time. In the further work, we will implement our proposed algorithm based on GPU.

## 4. Material Used in this Study

This study is a part of the project: “The Intelligent Farrowing Pen”, financed by the “Danish Advanced Technology Foundation”. The aim of this project was to develop an IT management and surveillance system for sows in farrowing pens. The system should be able to regulate the climate at farrowing pen level according to the animals’ actual needs and notify the farmer of any critical situation for sows and piglets in the farrowing pens.

All videos used were recorded at the experimental farm of Research Center Foulum, Denmark. The farrowing house (consisting of a total of 24 farrowing pens) was illuminated with common TL-lamps (*i.e*., Fluorescent lamps) which were hung on the ceiling, and the room lightning was always turned on. In each pen, a monochrome CCD camera (Monacor TVCCD-140IR) was fixed and positioned in such a way that the platform was located approximately in the middle of the farrowing pen. The cameras were connected to the MSH-Video surveillance system [35], which is a PC based video-recording system.

Two distinct data types were used: *training* and *test* data, which were recorded by MSH-Video surveillance system. The size of grayscale image (jpg) was 768×576. The training data sets were captured as 6 frames per minute, and the images in test data sets were captured as 1 frame per minute.

### 4.1. Training Data

The two data sets were recorded during two days in the same pen (about 8 h for every day, from 08.00 o’clock to 16.00 o’clock). The recordings took place after farrowing under varying illumination conditions. We used the training data sets to develop our algorithm.

In the beginning of the two sequences, about 200 consecutive images without the sow and piglets were captured with 10 s interval. In this initialization phase, for each data set, the light was often turned off/on in order to make it possible to update the background model in the Gaussian mixture model [36] without foreground objects under different lights. In this phase, the amount of lighting changes was about 15 times.

After about 40 min, the sow and piglets went into the pen and then the light was also often turned off/on. We made the light changes about 1 h after the sow and piglets went into the pen. In this period, the amount of lighting changes was about 30 times.

In the recordings, the nesting materials (*i.e*., straws) were moved around by the sow and piglets, and the sow were not moving over a long time. The following three problems were identified in the test data sets: sudden illumination changes, motionless foreground objects and dynamic background.

### 4.2. Test Data

The test data sets: ten data sets were randomly selected and recorded before farrowing (*i.e*., without piglets) from 0.00 o’clock to 24.00 o’clock in 6 different pens (one day for each set). They are used to analyse the behaviour of sows under different treatments.

### 4.3. Evaluation Criteria

In order to test the effectiveness of our algorithm, we had made the following criteria to evaluate the original and segmented images.

1. Criteria for original images:
  We manually evaluated the area of the sow for all original images in the test data and some original images in the training data. The evaluated area was used to compare with the corresponding shape of the sow in the segmented binary image.
  To demonstrate the segmentation efficiency under different illumination conditions, the original images were evaluated within about 1 hour in the two training data sets after the sow and piglets went into the pen, since the two periods contained different light conditions that were manually made. The original images were manually classified into two illumination levels: *Normal* and *Change*. The *Normal* level means that the lights were not or slowly changed and the *Change* level means that the lights were changed (*i.e*., the lights were switched on or off at that moment).
2. Criteria for segmented images:
  The segmented images were visually evaluated and classified into three scale groups. (1) Full Segmentation (*FS*): the shape of the sow was segmented over 90% of the manually evaluated area; (2) Partial Segmentation (*PS*): the shape of the sow was segmented between 80% and 90% of the manually evaluated area; (3) Cannot Segment (*CNS*): (a) there were two or more separated regions; (b) there were many false foreground pixels in the segmented image; (c) the shape of the sow was segmented below 80% of the manually evaluated area.
  The classification was based on a comparison (*i.e*., ratio) between the manually evaluated area and the corresponding area of the shape of the sow in the segmented image. The corresponding segmented images that represent the three scale groups are shown in Figure 7.

## 5. Experimental Results

### 5.1. HWF Evaluation

To evaluate our HWF, the mean square error (MSE) and peak-signal-to-noise ratio (PSNR) are chosen. The measured results for our HWF are compared to the measurements of the homomorphic filtering with the Butterworth high-pass filter (BHPF). The best filter must give its performance high in PSNR value and low MSE value [37]. The BHPF is given by [13]:

(20) $$h\left(\omega \right)=g_{l}+\frac{g_{h}-g_{l}}{1+{\left(d_{0}/\sqrt{\omega_{1}^{2}+\omega_{2}^{2}}\right)}^{2n}}$$

where *ω* is a complex frequency variable, n=2 is the order of the filter, $d_{0}=0.95$ is a cutoff frequency; $g_{h}=2.0$ and $g_{l}=0.5$ are maximal and minimal amplitudes of the BHPF corresponding to very high and low frequencies, respectively.

The MSE is the cumulative squared error between the filtered and original images, whereas PSNR is a measure of the peak error. The MSE and PSNR are given by:

(21) $$\mathrm{MSE}=\frac{1}{mn}\sum_{x=1}^{m}\sum_{y=1}^{n}{\left[f\left(x,y\right)-\tilde{f}\left(x,y\right)\right]}^{2},\;\mathrm{PSNR}=20\cdot \log_{10}\left(\frac{255}{\sqrt{\mathrm{MSE}}}\right)$$

where *f* and $\tilde{f}$ are the original and filtered images, respectively. The size of the image is m×n. The filtered image ${\tilde{f}}_{\mathrm{HWF}}$ for our proposed HWF is given ${\tilde{f}}_{\mathrm{HWF}}=\exp\left(\tilde{I}\right)$, where $\tilde{I}$ is given in Equation (6). The filtered image ${\tilde{f}}_{\mathrm{BHPF}}$ (*i.e*., the transforming back to image intensity space) is gotten by using the homomorphic filtering with the BHPF.

**Figure 3:**
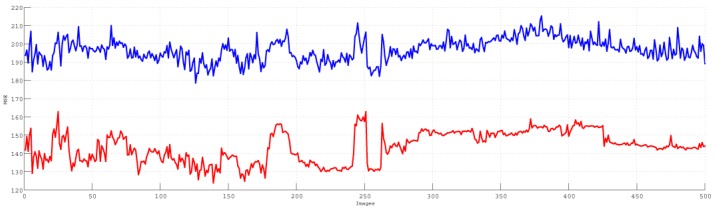
Mean square error (MSE) values for our homomorphic wavelet filter (HWF) and the filtering Butterworth high-pass filter (BHPF). The red line is for the HWF and the blue line is for the BHPF. The 500 consecutive images from our training data are selected in this evaluation.

**Figure 4:**
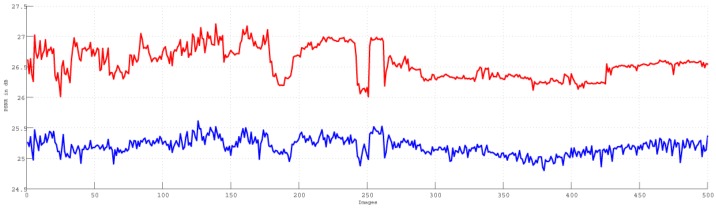
Peak-signal-to-noise ratio (PSNR) values for our HWF and the filtering BHPF. The red line is for the HWF and the blue line is for the BHPF. The 500 consecutive images from our training data are selected in this evaluation.

We randomly chose 500 consecutive images from the training data to compare our proposed HWF and the filtering BHPF. The comparison for MSE is shown in Figure 3. The red and blue lines show the MSE values for our HWF and the filtering BHPF, respectively. The comparison for PSNR is shown in Figure 4. The red and blue lines show the PSNR values for our proposed HWF and the filtering BHPF, respectively. The comparisons demonstrate that our HWF gives a better result than the BHPF.

### 5.2. WQI Evaluation

**Figure 5:**
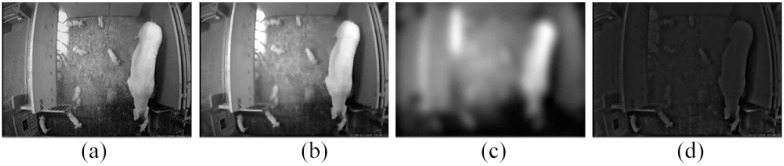
The wavelet quotient image (WQI) model evaluation: (**a**) the original image; (**b**) the diffusion image; (**c**) the coarse image; (**d**) the wavelet-quotient image (*i.e*., the reflectance image).

Figure 5 illustrates visually an example for the diffusion, coarse and wavetlet-quotient images which are presented in Section 2.2. Figure 5d is the reflectance component, which is estimated by our proposed WQI model.

### 5.3. Detection Evaluation

We both qualitatively and quantitatively evaluate the segmented images in this subsection. The evaluation criteria for three scale groups of segmented images is described in Section 4.3.

#### 5.3.1. Qualitative Evaluation

**Figure 6:**
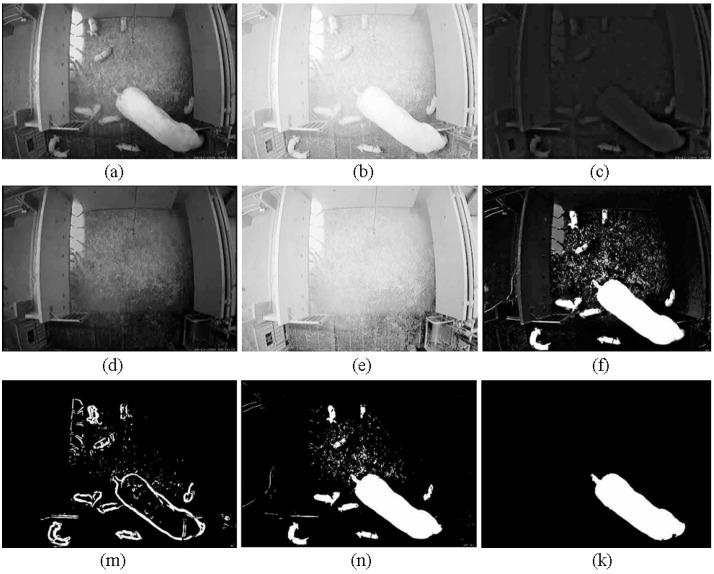
The general segmented result under light changes in the training data: (**a**) the current image; (**b**) the estimated illumination of the current image in logarithmic space; (**c**) the estimated reflectance of the current image in intensity space; (**d**) the reference image; (**e**) the estimated illumination of the reference image in logarithmic space; (**f**) the synthesized image; (**m**) the boundaries of the foreground objects; (**n**) the binary image after *k*-means on the synthesized image (*i.e.*, image f); (**k**) the foreground object (*i.e*., the sow) is obtained by using the images m and n.

Figure 6 represents an example of the general segmented results under varying illumination conditions in the training data. Obviously, the synthesized image (*i.e*., Figure 6f) shows that the homogeneous regions are accurately classified as either foreground objects or background objects by using Equation (15).

In our application, there were some nesting materials (e.g., straws) in the farrowing pen, which were often moved by the sow and the piglets. In these regions, the background was dynamic, the coarse coefficients of the dynamic background could be relatively regularized by the DWT, and the coarse coefficients were used in our HWF to estimate the illumination component. This means that the dynamic background became a relatively static background by the HWF. This demonstrates that our approach is less sensitive to the dynamic background. Since the sow and piglets in the current image were the actual motions w.r.t. the reference image, therefore, a foreground object in the current image that becomes motionless can be distinguished from the background objects.

**Figure 7:**
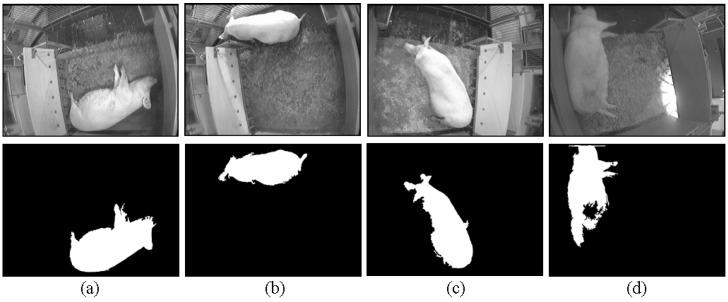
The general segmented results of the scale groups in the test data: (**a**) *FS*: under slow changes of illumination; (**b**) *FS*: under sudden changes of illumination; (**c**) *PS*; (**d**) *CNS*.

Figure 7 represents the general segmented images of the three scale groups in the test data. The segmented images in *FS* and *PS* scale groups can be used to track the simple behaviour of sows, such as their position (*i.e*., center of mass of the segmented sow shape) and orientation (*i.e*., the angle between the x-axis and the major axis of the ellipse (*i.e*., the sow shape)). As shown in Figure 7d, the most segmented images in *CNS* scale group are because the sows are dirty with caked mud and manure (*i.e*., the caked mud and manure on the sow). In fact, the caked mud and manure are the background objects in our application.

#### 5.3.2. Quantitative Evaluation

In addition to the qualitative evaluation, we provide a quantitative evaluation. Table 2 shows the results of the evaluation for the segmented images in the test data. Over 98.5% of the segmented images can be used to track the behaviour of sows.

**Table 2:** The segmented binary images are manually classified into the three groups in the test data. *FS*: Full Segment; *PS*: Partial Segment; *CNS*: Cannot Segment. N: number of images; %: percentage of total images.

Data Set	Total Images		*FS*			*PS*			*CNS*	
			N	%		N	%		N	%
1	1435		1372	95.61		43	2.99		20	1.39
2	1434		1370	95.54		46	3.21		18	1.26
3	1437		1369	95.27		51	3.55		17	1.18
4	1435		1374	95.75		42	2.93		19	1.32
5	1436		1375	95.75		41	2.86		20	1.39
6	1435		1367	95.26		43	2.30		25	1.74
7	1436		1369	95.33		45	3.13		22	1.53
8	1433		1372	95.74		41	2.86		20	1.40
9	1438		1375	95.19		48	3.34		15	1.04
10	1435		1369	95.40		43	3.00		23	1.63
Average				95.48			3.02			1.39

### 5.4. Comparison

In order to demonstrate that our algorithm can efficiently detect the foreground objects, we compare our algorithm with the Gaussian mixture model (GMM) [36] by using the training data.

In the GMM-based method, the first 200 images (without foreground objects, see Section 4.1) of each data set were the recent history data for the GMM-based method. The foreground objects $f_{c}^{fg}$ of the current image that are detected by using the GMM-based method combines with the foreground boundaries $f_{c}^{bou}$ of the current image which are calculated by Equation (19). The combination between the two binary images $f_{c}^{fg}$ and $f_{c}^{bou}$ is presented in Section 2.5.

We manually evaluated the first 360 (with foreground objects, *i.e*., the sow and piglets went into the pen) original and segmented images of each set in the training data, since the two periods contained light changes which were manually made. The evaluation criteria for two levels of lights and three scale groups of segmented images is described in Section 4.3.

Table 3 shows the manually evaluated results. For the *Change* level, the rates of *CNS* for our algorithm and GMM-based method are 2.4% and 82.5%, respectively. This evaluation demonstrates that our algorithm is able to deal with sudden light changes.

**Figure 8:**
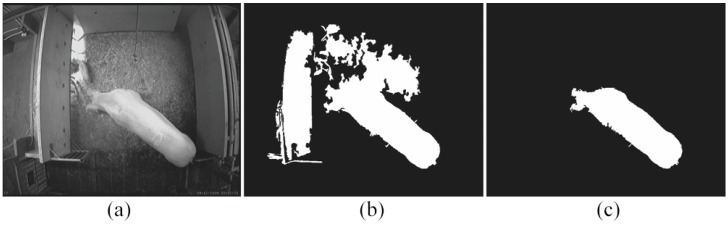
Sudden illumination changes: (**a**) the original image; (**b**) the segmented result based on the GMM model; (**c**) the segmented result by using our algorithm. The example shows that the Gaussian mixture model (GMM)-based method fails under sudden light change.

Figure 8 gives an example while the GMM-based method fails under the sudden illumination change, but the foreground objects can still be successfully detected by our algorithm.

**Table 3:** The visual evaluation for the light changes by using 720 images in the training data sets. The original images are classified into two illumination levels: *Normal* and *Change*. The corresponding segmented binary images are manually classified into three groups. *FS*: Full Segment; *PS*: Partial Segment; *CNS*: Cannot Segment; N: number of images; %: percentage of total images.

Illumination	N	Our Algorithm						GMM-Based Method					
		*FS*		*PS*		*CNS*		*FS*		*PS*		*CNS*	
Level		N	%	N	%	N	%	N	%	N	%	N	%
*Normal*	594	589	99.15	4	0.67	0.17	0.27	544	91.58	42	7.07	8	1.34
*Change*	126	117	92.9	6	4.7	3	2.4	9	7.2	13	10.3	104	82.5

In order to evaluate the performance of our algorithm more quantitatively, we compare the receiver operating characteristic (ROC) curves between the algorithm and the GMM-based method. The true positive fraction (TPF) and the false positive fraction (FPF) that construct a ROC curve are calculated as following:

(22) $$\mathrm{TPF}=\frac{\mathrm{FP}}{\mathrm{FP}+\mathrm{TN}}\;\mathrm{and}\;\mathrm{FPF}=\frac{\mathrm{TP}}{\mathrm{TP}+\mathrm{FN}}$$

where FP (false positive) indicates pixels falsely marked as the foreground, TN (true negative) the number of correctly identified background pixels, TP (true positive) the number of correctly detected foreground pixels, FN (false negative) pixels falsely marked as the background.

**Figure 9:**
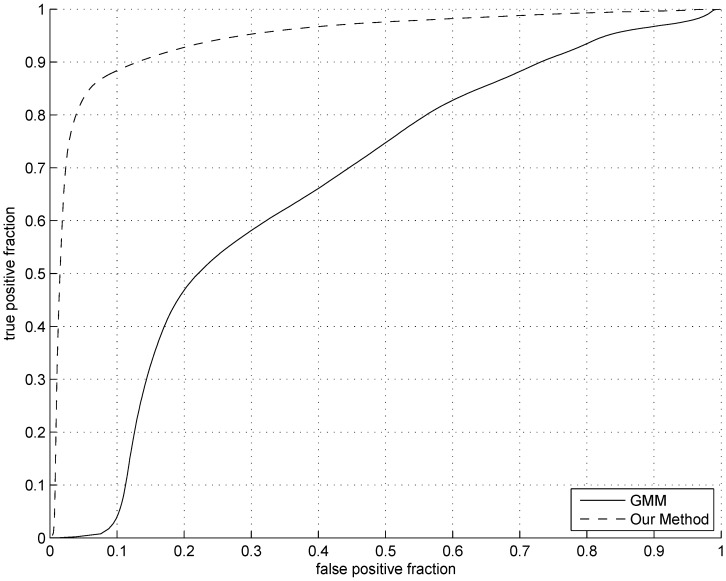
The receiver operating characteristic (ROC) curves: the dashed line is ROC curve of our method, the other one is ROC curve of the GMM-based method.

We have randomly selected 12 images to plot the ROC curve. The selected 12 images were classified into the *Change* level in Table 3: 9 images were randomly chosen from the *FS* group, 2 images were randomly chosen from the *PS* group and 1 image was randomly chosen from the *CNS* group. For example, the image in Figure 8 was one of our choices. The corresponding original image was manually segmented to generate the ground-truth (only the sow). Figure 9 shows the comparison of two ROC curves of our algorithm and GMM-based method.

### 5.5. The Publicly Available Data

We also apply our algorithm on a publicly available dataset: /dataset2014/thermal/library to detect the foregrounds. The dateset is downloaded from the website: http://changedetection.net and has two main parts: the original frames and their corresponding ground truth. It contains 4900 frames of frame size 320 x 240. Since the beginning and ending frames are the background frames in the dataset, therefore 3340 frames (*i.e*., the frame numbers ranged from 860 to 4200) are selected to detect the foreground objects.

In order to detect the foreground objects, the original frames (colour) are converted to grey images and then the first 7 steps of our algorithm (see Table 1) are performed, *i.e*., the foreground objects are extracted on the synthesizing image (*i.e*., Equation (15)) by using Equation (18).

**Figure 10:**
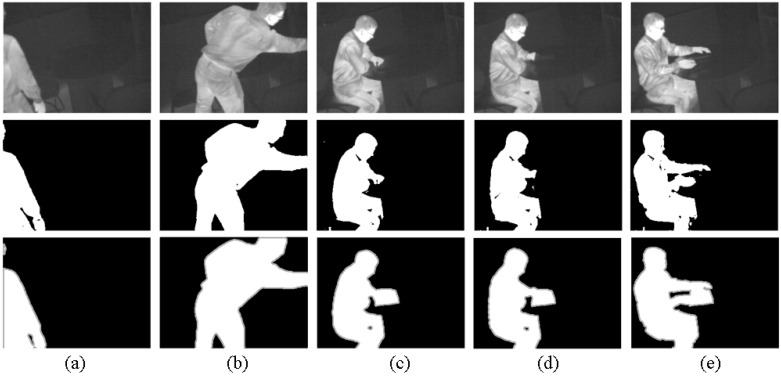
The general segmented result in the publicly available data: first row - the original frames; second row-our results; third row-the ground truths. Frame number in the dataset: (**a**) number 904; (**b**) number 1030; (**c**) number 2709; (**d**) number 3340; (**e**) number 4118.

Figure 10 shows the general segmented results. The precision is 0.9939 for the 3340 frames and defined as the proportion of the TP against all the positive results (both TP and FP):

(23) $$\mathrm{Precision}=\frac{\mathrm{TP}}{\mathrm{TP}+\mathrm{FP}}$$

## 6. Conclusions

We have presented an approach to estimate the illumination and reflectance components of an image. A homomorphic wavelet filter is used to estimate the illumination component. A wavelet-quotient image model which is used to estimate the reflectance component is defined.

Based on the approach, we have developed an algorithm to segment the sows in the complex farrowing pens. The experimental results have demonstrated that the algorithm can be applied to detect the domestic animals in complex environments such as light changes, motionless foreground objects and dynamic background.

The proposed approach is sensitive if the textures of foreground and background objects are very similar. Also, the high computational cost is the drawback of our approach and in the further work we will implement it using data parallel computing based on GPU.
